# AGL15 Controls the Embryogenic Reprogramming of Somatic Cells in Arabidopsis through the Histone Acetylation-Mediated Repression of the miRNA Biogenesis Genes

**DOI:** 10.3390/ijms21186733

**Published:** 2020-09-14

**Authors:** Katarzyna Nowak, Joanna Morończyk, Anna Wójcik, Małgorzata D. Gaj

**Affiliations:** Institute of Biology, Biotechnology and Environmental Protection, Faculty of Natural Sciences, University of Silesia in Katowice, 40-007 Katowice, Poland; joanna.moronczyk@us.edu.pl (J.M.); anna.wojcik@us.edu.pl (A.W.); malgorzata.gaj@us.edu.pl (M.D.G.)

**Keywords:** somatic embryogenesis, AGL15, miR156, miRNA biogenesis, acetylation, HDAC, TOPLESS co-repressor, HEN1, SERRATE, DCL1

## Abstract

The embryogenic transition of somatic cells requires an extensive reprogramming of the cell transcriptome. Relevantly, the extensive modulation of the genes that have a regulatory function, in particular the genes encoding the transcription factors (TFs) and miRNAs, have been indicated as controlling somatic embryogenesis (SE) that is induced in vitro in the somatic cells of plants. Identifying the regulatory relationships between the TFs and miRNAs during SE induction is of central importance for understanding the complex regulatory interplay that fine-tunes a cell transcriptome during the embryogenic transition. Hence, here, we analysed the regulatory relationships between AGL15 (AGAMOUS-LIKE 15) TF and miR156 in an embryogenic culture of Arabidopsis. Both *AGL15* and miR156 control SE induction and AGL15 has been reported to target the *MIR156* genes *in planta*. The results showed that AGL15 contributes to the regulation of miR156 in an embryogenic culture at two levels that involve the activation of the *MIR156* transcription and the containment of the abundance of mature miR156 by repressing the miRNA biogenesis genes *DCL1* (DICER-LIKE1), *SERRATE* and *HEN1* (HUA-ENHANCER1). To repress the miRNA biogenesis genes AGL15 seems to co-operate with the TOPLESS co-repressors (TPL and TPR1-4), which are components of the SIN3/HDAC silencing complex. The impact of TSA (trichostatin A), an inhibitor of the HDAC histone deacetylases, on the expression of the miRNA biogenesis genes together with the ChIP results implies that histone deacetylation is involved in the AGL15-mediated repression of miRNA processing. The results indicate that HDAC6 and HDAC19 histone deacetylases might co-operate with AGL15 in silencing the complex that controls the abundance of miR156 during embryogenic induction. This study provides new evidence about the histone acetylation-mediated control of the miRNA pathways during the embryogenic reprogramming of plant somatic cells and the essential role of AGL15 in this regulatory mechanism.

## 1. Introduction

The molecular mechanisms that underlie the phenomenon of the toti-/pluripotency of somatic cells remain at the centre of developmental biology. In plants, insights into somatic embryogenesis (SE) that is induced in *in vitro*-cultured explants contribute to identifying the regulatory processes that control the embryogenic reprogramming of somatic cells. Transcription factors (TFs) and miRNAs play a central role in the response of a somatic cell transcriptome to the SE-inducing factors such as auxin treatment [1,2,3,4]. Hence, identifying the regulatory relationships between the TFs and miRNAs is of central importance for understanding the complex network that fine tunes the transcriptomes of the somatic cells during the induction of SE.

The SE-involved genes encode numerous TFs that have hormone- and stress-related functions [5,6,7,8,9] and among them, AGAMOUS-LIKE15 (AGL15) of the MADS-box family of TFs has been identified [10,11]. *AGL15* encodes the MADS-domain protein that selectively binds to a consensus DNA sequence, the CArG (C-A/T rich-G) motif, in order to either activate or repress the expression of the target genes [12]. The binding of AGL15 to numerous hormone-related loci in the Arabidopsis genome implies that AGL15 controls embryogenic induction by regulating gibberellic acid and ethylene metabolism and auxin signalling [13,14]. Accordingly, AGL15 directly targets the GIBBERELLIN 2-OXIDASE2, GIBBERELLIC ACID INSENSITIVE, and ACC SYNTHASE, ACC OXIDASE genes that regulate the metabolism of gibberellic acid and ethylene, respectively [15,16,17]. Accordingly, AGL15 might positively affect SE induction by directly activating GA 2-oxidase, which results in a decreased level of biologically active GA [10]. AGL15 also affects auxin signalling as a result of the repression of the TRANSPORT INHIBITOR RESPONSE 1, AUXIN RESPONSE FACTOR 6 and *IAA30* genes [14]. In addition to directly regulating the hormone-related genes AGL15 also indirectly affects hormone metabolism and signalling via regulatory interactions with other TFs that have been indicated as having essential functions in SE, including LEAFY COTYLEDON2, FUSCA3 and BABY BOOM (reviewed in [18,19,20]).

In addition to protein-encoded genes, the *MIR* genes encoding microRNA (miRNAs) [13,21] are also targets of AGL15. miRNAs, which are small (19–24 nucleotide long), single-stranded non-coding RNA molecules, regulate both the state of the chromatin which is associated with target genes and the availability of the encoded transcripts for protein translation [22]. In the control of plant development, miRNAs preferentially target the genes that have a regulatory function including those encoding the TFs [23,24].

The differential expression of numerous miRNAs, mostly hormone-related, has been attributed to SE induction in different plants including Arabidopsis [25,26,27,28,29,30]. Auxin-related functions during SE induction have been demonstrated for miR393, miR396, miR160 and miR167, which have an auxin-related function [31,32,33]. An analysis of the expression of different *MIR*s in embryogenic cultures of Arabidopsis and other plants has indicated highly diverse expression patterns between the members of the same *MIR* gene family [27,30,34,35,36]. Therefore, the extensive transcriptional regulation of different *MIR* genes seems to contribute to the production of functional miRNA molecules during SE induction. Although the *MIR* promoters, similar to the protein-coding genes might be activated or repressed by the binding of specific regulatory proteins, only a few TFs that target specific *MIR* genes have been identified to date [37].

The contribution of AGL15 to the positive regulation of the *MIR156* genes has been reported during flower development [21]. Importantly, *MIR156* genes were reported to be among the potential targets of AGL15 in an embryogenic culture [13] and consequently, the regulatory functions of miR156 in SE induction in different plants have been postulated (reviewed in [1,4]). Moreover, the accumulation of both *AGL15* and miR156 transcripts in the cotyledons of Arabidopsis that contribute to SE induction in this plant has been demonstrated [21,38]. Taken together, these findings suggest that AGL15 might control miR156 during SE induction.

The regulatory mechanism by which AGL15 might control the target genes involves interactions with the SIN3/HDAC (Swi-independent3/Histone Deacetylase) silencing complex [39]. By recruiting the TOPLESS (TPL) and TPL-RELATED (TPR) transcriptional corepressors [40], AGL15 directs the histone deacetylases (HDACs) to the target gene loci [41]. The HDACs that operate in the SIN3/HDAC silencing complex include HDAC19 and HDAC6 of the Reduced Potassium Dependence 3/Histonee Deacetylase 1 family of histone deacetylases whose members play a critical role in various development processes and stress responses (reviewed in [42]). The function of HDAC6 and HDAC19 in controlling the embryogenesis-related genes during zygotic embryogenesis has been documented including the HDAC19-mediated repression of *LEC1* and *LEC2* genes, which are the master regulators of both zygotic and somatic embryogenesis [20,43,44,45]. Relevantly, although HDAC6 and HDAC19 have been postulated to function in the histone acetylation-mediated control of SE, the target genes remain to be revealed [40,42,46,47].

Here, in order to verify the assumption about the regulatory relationship of *AGL15* and the abundance of miR156 in SE, we analysed the impact of AGL15 on both the transcription of the *MIR156* genes and the level of mature miR156 molecules in an embryogenic culture of Arabidopsis. Insights into the gene expression profiles in different mutants and transgenic lines together with the results of the ChIP analysis indicated that AGL15 contributes to the regulation of miR156 in an embryogenic culture at two levels that involve the activation of *MIR156* transcription and the containment of the abundance of mature miR156 by repressing the miRNA biogenesis genes *DCL1*, *SERRATE,* and *HEN1*. We also showed that to repress the miRNA biogenesis genes AGL15 seems to co-operate with other components of the SIN3/HDAC silencing complex including the TOPLESS co-repressors and HDAC6 and HDAC19 histone deacetylases. The study provides new evidence about the histone acetylation-mediated control of the miRNA pathways during the embryogenic reprogramming of plant somatic cells and the essential role of *AGL15* in this regulatory mechanism.

## 2. Results

### 2.1. AGL15 Impacts the Abundance of Mature miR156

**Figure 1:**
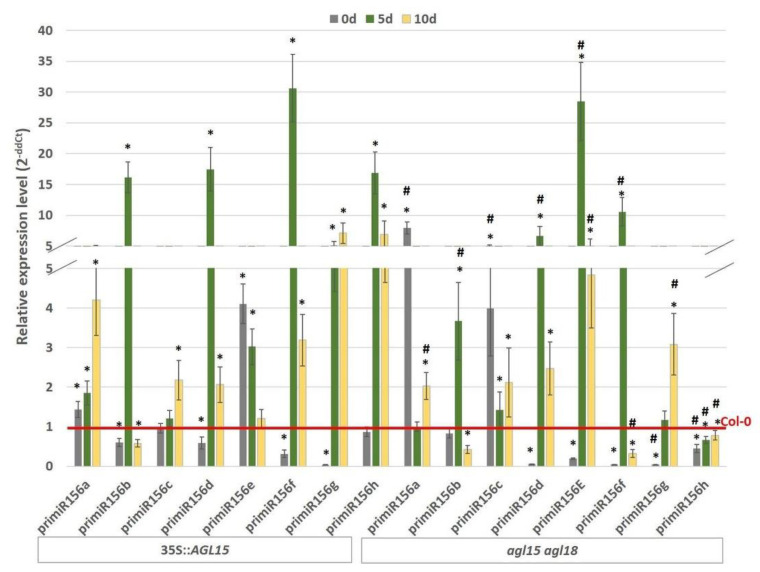
Relative expression level of *pri-miR156s* in the embryogenic cultures of the 35S::*AGL15* and *agl15 agl18* transgenic lines. The relative miRNA level was normalised to an internal control (*At4g27090*) and calibrated to a Col-0 culture of the same age. * value significantly different from the Col-0 culture of the same age (*p* < 0.05; *n* = 3 ± SD); ^#^ value significantly different from the 35S::*AGL15* culture of the same age (*p* < 0.05; *n* = 3 ± SD).

The role of AGL15 in the activation of two *MIR156* genes (*MIR156a* and *c*) during flowering [21] motivated us to gain insights into the regulatory relationship between *AGL15* and miR156 in SE induction. To this end, the abundance of the primary miR156 transcripts (pri-miR156) of eight *MIR156* (*MIR156a–h*) genes that are present in the Arabidopsis genome was analysed in cultures that had different *AGL15* expression levels, including WT (Col-0), 35S::*AGL15,* and *agl15 agl18*. The results showed that there was no relationship between the *AGL15* expression level and the transcription of seven of the *MIR156* genes (*MIR156a–g*) and that both the overexpression (35S::*AGL15*) and knock-out mutation (*agl15 agl18)* resulted in a significant increase in pri-miR156a-g (Figure 1). By contrast, pri-miR156h had a high (up to 15-fold) up-regulation in response to the overexpression of *AGL15* and a distinct downregulation (up to two-fold) in the *agl15agl18* mutant culture, which suggests that AGL15 might positively regulate *MIR156h* during SE induction.

**Figure 2:**
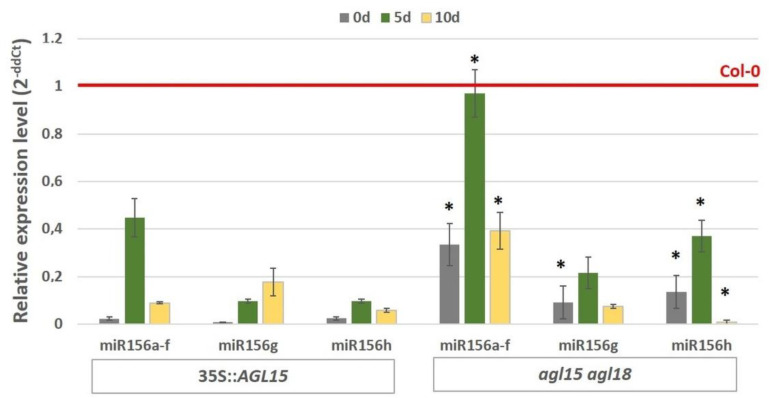
Abundance of mature miR156s in the embryogenic cultures of the 35S::*AGL15* and *agl15 agl18* transgenic lines. The relative miRNA level was normalised to an internal control (*At4g27090*) and calibrated to the Col-0 culture of the same age. * value significantly different from the 35S::*AGL15* culture of the same age (*p* < 0.05; *n* = 3 ± SD).

To learn more about the role of AGL15 in regulating miR156 during embryogenic induction, we analysed the accumulation of mature miR156 compared to the *AGL15* expression level. Unexpectedly, the results showed that AGL15 significantly modulated the abundance of mature miR156 and that there was a distinctly higher (from 2- to 14-fold) abundance of mature miR156 in the *agl15 agl18* mutant than in the 35S::*AGL15* culture (Figure 2). The results suggest a repressive function of AGL15 in the processing of miR156 molecules that precedes the production of functional miR156 molecules during the embryogenic reprogramming of a somatic cell.

### 2.2. AGL15 Negatively Controls the Expression of the miRNA Biogenesis Genes during Embryogenic Induction

**Figure 3:**
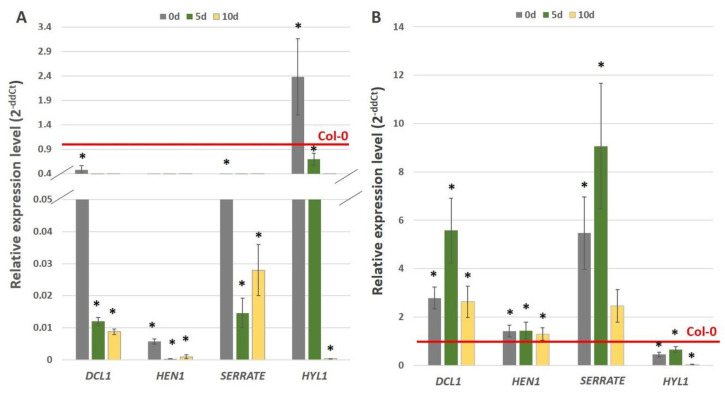
Relative expression level of the miRNA biogenesis genes (*DCL1*, *HEN1*, *SERRATE* and *HYL1*) in the embryogenic cultures of the 35S::*AGL15* (**A**) and *agl15 agl18* (**B**) transgenic lines. The relative expression level was normalised to an internal control (*At4g27090*) and calibrated to the Col-0 culture of the same age. * value significantly different from the Col-0 culture of the same age (*p* < 0.05; *n* = 3 ± SD).

The post-transcriptional mechanism that extensively fine tunes the mature miRNA level via miRNA-processing machinery has been assumed in an embryogenic culture [30]. Thus, we hypothesised that AGL15 might interact with the components of the miRNA biogenesis pathway to control the production of the functional mature miR156 during SE induction. To verify this assumption, we determined that it was reasonable to assess the regulatory relationships between *AGL15* and the genes encoding the critical components of miRNA biogenesis genes including DICER-LIKE1 (*DCL1*), *SERRATE*, HUA-ENHANCER1 (*HEN1*) and HYPONASTIC LEAVES1 (*HYL1*). To this end, the expression of these genes relative to the *AGL15* expression level was evaluated in embryogenic cultures of the 35S::*AGL15* and *agl15 agl18* double-mutant lines. The analysis indicated a significant decrease (2- to more than 3000-fold) in the *DCL1*, *SERRATE*, *HEN1* and *HYL1* expression in the SE culture that overexpressed *AGL15* (Figure 3A) and the upregulation of *DCL1*, *SERRATE*, *HEN1* in the culture of the *agl15 agl18* mutant (Figure 3B). By contrast, the expression of *HYL1* was downregulated in the both 35S::*AGL15* and *agl15 agl18* cultures compared to WT (Col-0), which implies a lack of any regulatory impact of AGL15 on *HYL1*. In summary, the results suggest that AGL15 may negatively affect the expression of three of the miRNA biogenesis genes including *DCL1*, *HEN1,* and *SERRATE.*

### 2.3. Histone Acetylation Might Be Involved in the AGL15-Mediated Control of the miRNA Biogenesis Genes in SE Induction

**Figure 4:**
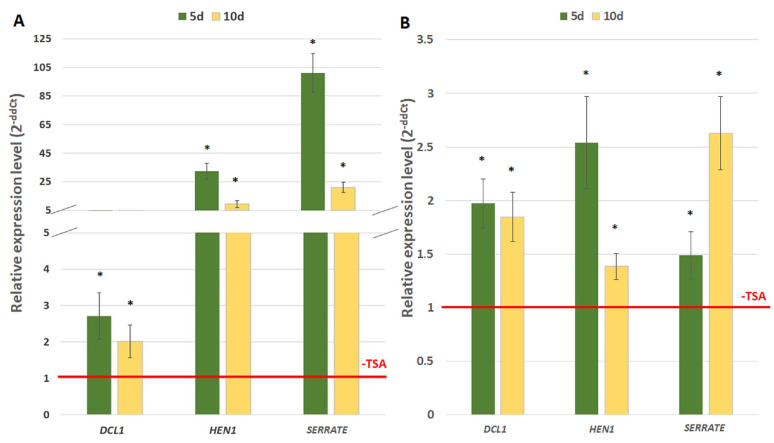
Relative expression level of the miRNA biogenesis genes (*DCL1*, *HEN1, SERRATE*) in the embryogenic cultures of the 35S::*AGL15* (**A**) and *agl15 agl18* (**B**) transgenic lines that had been treated with a TSA (Trichostatin A). The relative expression level was normalised to an internal control (*At4g27090*) and calibrated to the untreated culture of the genotypes of the same age. * value significantly different from the culture of the same age untreated with a TSA (*p* < 0.05; *n* = 3 ± SD).

The possible mechanisms that are involved in the AGL15-mediated repression of the target genes might include the interaction of AGL15 with a SIN3/HDAC silencing complex by the histone deacetylases, HDACs [48]. Therefore, we hypothesised that AGL15 acts as a repressor of the miRNA biogenesis genes (*DCL1*, *HEN1*, and *SERRATE*) in embryogenic culture via its interaction with the HDACs. To verify this hypothesis, we evaluated the effect of the HDAC inhibitor, trichostatin A (TSA), on the *DCL1*, *SERRATE*, and *HEN1* gene expression in the 35S::*AGL15* and *agl15 agl18* mutant cultures. We found that there was a significant up-regulation (from 2- to 120-fold) of the *DCL1*, *SERRATE* and *HEN1* genes in the culture that was overexpressing *AGL15* and a distinctly smaller increase of the gene transcripts in the mutant culture (Figure 4). This result supported the assumption of the role of histone acetylation in the AGL15-mediated control of the miRNA biogenesis gene expression during SE induction.

**Figure 5:**
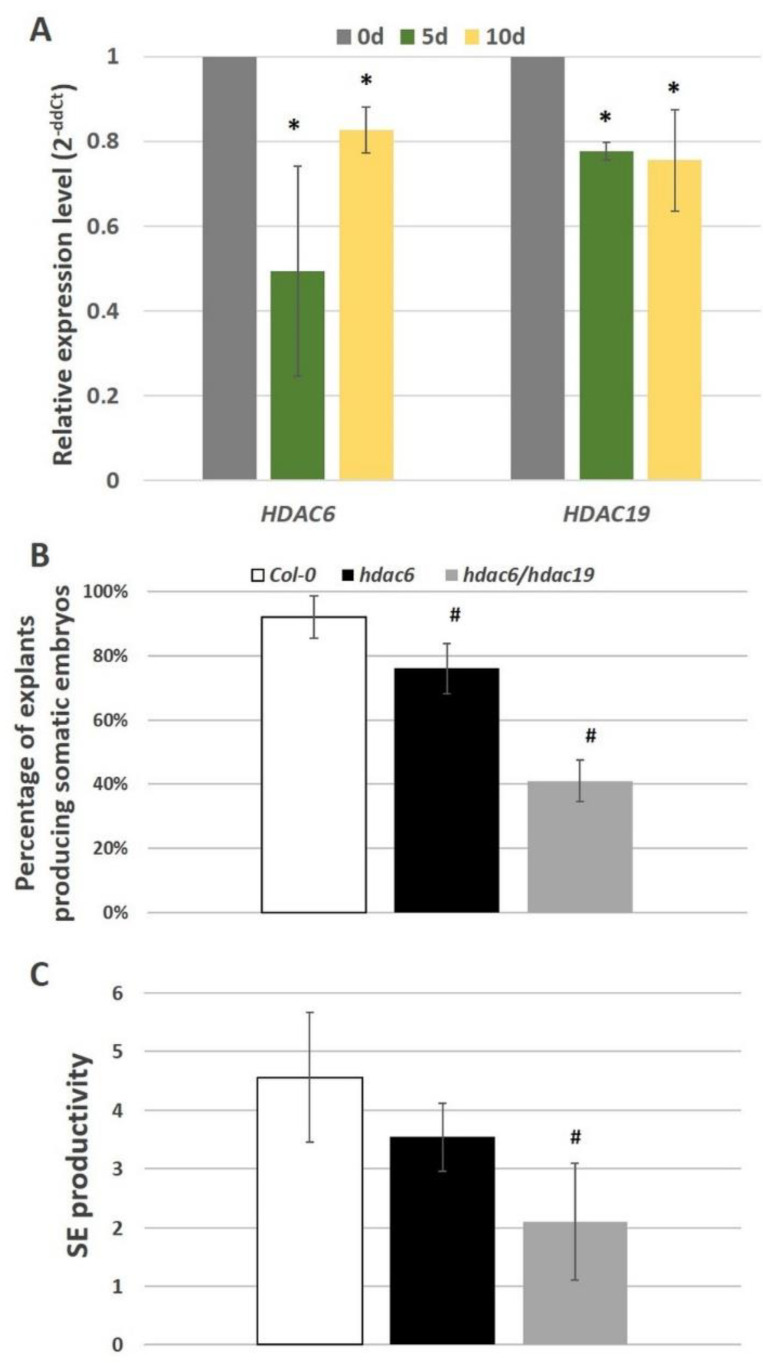
Expression analysis of *HDAC6* and *HDAC19* (**A**) in the SE culture of the Col-0 genotype. Capacity for SE in the cultures of the *hdac6* and *hdac6 hdac19* mutants and their parental genotype, Col-0. Percentage of explants producing somatic embryos (**B**) and SE productivity (**C**) of the IZE explant culture that was induced on an E5 medium. The relative transcript level was normalised to an internal control (*At4g27090*) and calibrated to freshly isolated explants (0d). * value significantly different from the freshly isolated explants (0d) (*p* < 0.05; *n* = 3 ± SD); ^#^ values significantly different from the Col-0 (*p* < 0.05; *n* = 3 ± SD).

**Figure 6:**
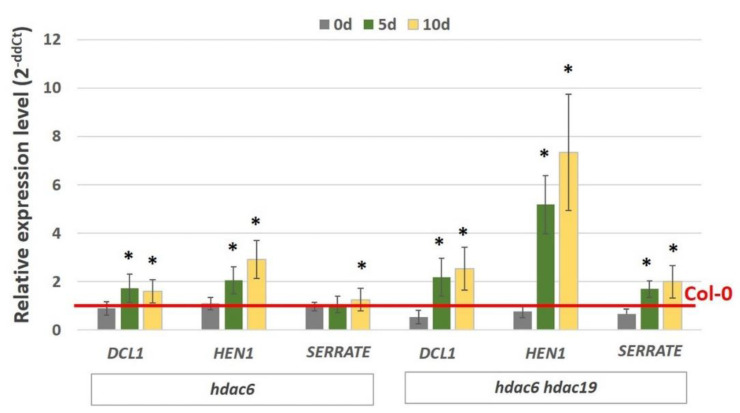
Relative expression of the miRNA biogenesis genes (*DCL1*, *HEN1*, *SERRATE*) in the SE cultures of the *hdac6* and *hdac6 hdac19* mutant lines. The relative transcript level was normalised to an internal control (*At4g27090*) and calibrated to the Col-0 culture of the same age. * values significantly different from the Col-0 culture of the same age (*p* < 0.05; *n* = 3 ± SD).

Next, we examined whether the histone deacetylases HDAC6 and HDAC19, which have a regulatory interaction with the AGL15 protein [48], might contribute to the AGL15-mediated control of the miRNA biogenesis genes in an embryogenic culture. Relevantly, we found that the *HDAC6* and *HDAC19* genes were downregulated in the embryogenic culture (Figure 5A) and that the mutants that were defective in these genes (*hdac6* and *hdac6/hdac19*) had a significantly impaired embryogenic response (Figure 5B,C). These results implied a role of *HDAC6* and *HDAC19* in SE induction. Next, to verify whether HDAC6 and HDAC19 might control embryogenic induction via the regulation of miRNA biogenesis genes, we analysed the expression of *DCL1*, *HEN1*, and *SERRATE* in the embryogenic culture of *hdac6* and *hdac6 hdac19* mutants. The analysis indicated that the impaired expression of *HDAC6/19* in the mutants resulted in an increase in the expression of *DCL1*, *HEN1*, and *SERRATE* during somatic embryogenesis (Figure 6). The results provide support for the role of *HDAC6/19* in the histone acetylation-mediated control of miRNA biogenesis during embryogenic induction.

### 2.4. AGL15 Impacts the Acetylation Level of Histone H3, Which Is Associated with the Promoter of the DCL1 and SERRATE Genes in an Embryogenic Culture

The results suggest that in an embryogenic culture, AGL15 might repress the expression of the miRNA biogenesis genes *DCL1*, *HEN1* and *SERRATE*, via histone deacetylation. To confirm this assumption, we examined the histone acetylation in the chromatin that is associated with the promoters of the miRNA biogenesis genes during embryogenic induction. The H3K9 and K14 positions, which have a positive influence on gene expression [49,50] were analysed. Most of the AGL15-bound sites are located within 1 kb of the transcription unit of the target gene and carry at least one CArG motif [13]. It was observed that the fragments of *DCL1*, *SERRATE* and *HEN1* promoters that were analysed using ChIP were located upstream (−600 bp or −400 bp) and downstream (+300 bp) to TSS and included one to six CArG sequences (Supplementary Figure S1). ChIP analysis using the anti-H3K9/14ac antibody was performed and the different promoter regions that include the TSS + 300 and CArG sequences were examined in the explants of the 35S::*AGL15* and *agl15 agl18* transgenic lines that had been induced for five days on an SE-induction medium.

**Figure 7:**
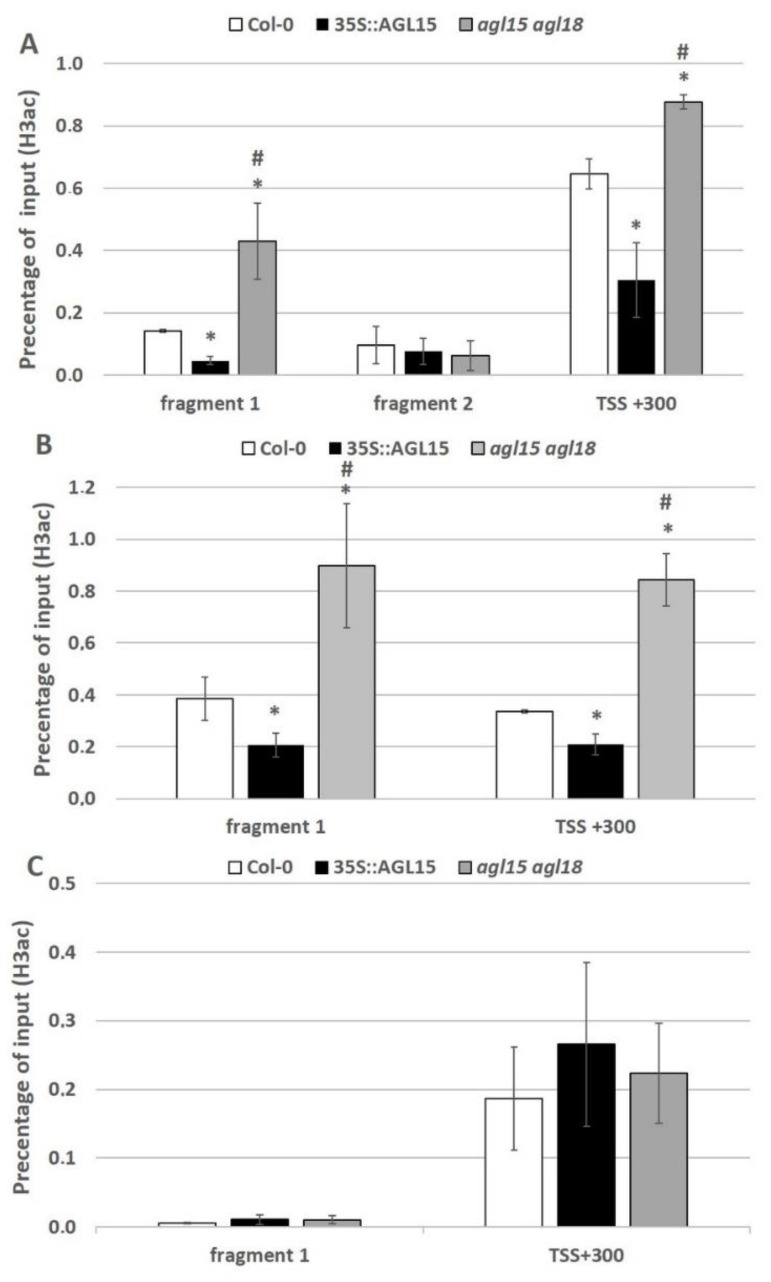
H3ac enrichment in a selected fragment of a promoter and TSS + 300 of the *DCL1* (**A**), *SERRATE* (**B**) and *HEN1* (**C**) genes in the explants of the Col-0, 35S::*AGL15* and *agl15 agl18* lines. * value significantly different from the Col-0 (*p* < 0.05; *n* = 3 ± SD); ^#^ value significantly different from the 35S::*AGL15* (*p* < 0.05; *n* = 3 ± SD).

The analysis revealed AGL15-dependent changes in H3 acetylation in the chromatin which is associated with the analysed regulatory fragments (fragment 1 and TSS + 300) of two genes *DCL1* and *SERRATE* (Figure 7). The acetylation level of histone H3 associated with these genes was substantially increased and decreased in the *agl15 agl18* mutant and the *AGL15* overexpression line cultures, respectively. In conclusion, AGL15 might negatively control the miRNA biogenesis genes *DCL1* and *SERRATE* during SE induction via histone acetylation-related mechanisms in which HDAC6 and HDAC19 seem to operate.

### 2.5. TPL Co-Repressors Might Contribute to the AGL15-Mediated Control of the miRNA Biogenesis Genes in an Embryogenic Culture

**Figure 8:**
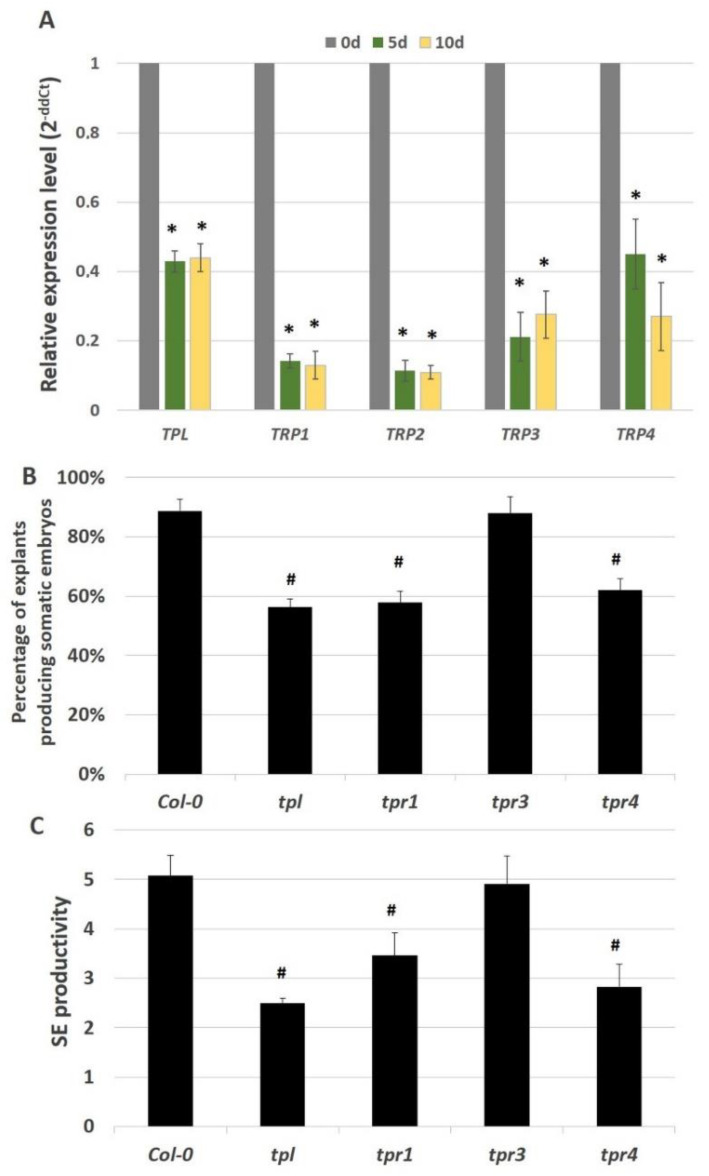
Relative expression level of the genes encoding the TOPLESS co-repressors (*TPL*, *TPR1-4*) in the embryogenic culture of Col-0 (**A**). Capacity for somatic embryogenesis in the cultures of mutants in the TOPLESS co-repressor genes (*tpl, tpr1, 3, 4*) and their parental genotype, Col-0. Percentage of explants producing somatic embryos (**B**) and SE productivity (**C**) of the IZE explant culture that was induced on an E5 medium. The relative expression level was normalised to an internal control (*At4g27090*) and calibrated to freshly isolated explants (0d). * value significantly different from the freshly isolated explants (0d) (*p* < 0.05; *n* = 3 ± SD); ^#^ values significantly different from Col-0 (*p* < 0.05; *n* = 3 ± SD).

To identify any other elements of the AGL15-related complex that silence the expression of the miRNA biogenesis genes in somatic embryogenesis, we investigated the possible involvement of the TOPLESS (TPL) co-repressors, which directly interact with AGL15 [39]. The expression profiling of the *TPL* and four *TPR* (*TPR1-4*) genes indicated that they were significantly down-regulated during embryogenic induction (Figure 8A). Together with the severely impaired embryogenic potential of the *tpl*, *tpr1,* and *tpr4* mutant culture (Figure 8B,C), the results suggest a role of the TPL/TPR co-repressors (*TPL*, *TPR1*, *TPR4*) in somatic embryogenesis induction.

**Figure 9:**
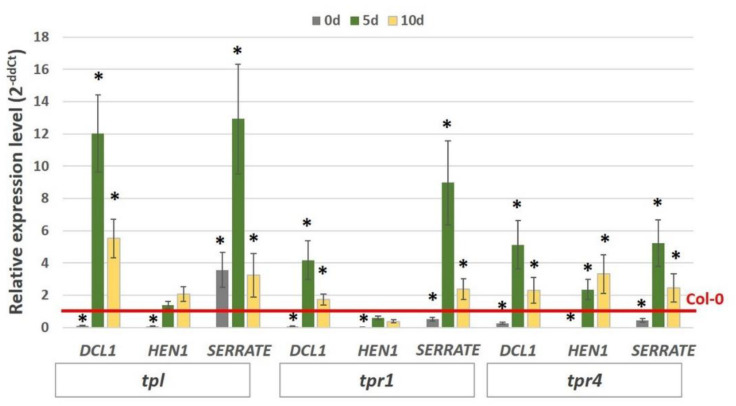
Relative expression of the miRNA biogenesis genes (*DCL1*, *HEN1*, *SERRATE*) in the embryogenic culture of the TOPLESS co-repressor mutants (*tpl*, *tpr1*, *tpr4*). The relative transcript level was normalised to an internal control (*At4g27090*) and calibrated to the Col-0 culture of the same age. * values significantly different from the Col-0 culture of the same age (*p* < 0.05; *n* = 3 ± SD).

Next, we assumed that the TPLs might impact the miRNA biogenesis genes in an embryogenic culture and the expression of *DCL1*, *HEN1,* and *SERRATE* in the *tpl*, *tpr1* and *tpr4* mutants was analysed in order to verify this hypothesis. The analysis showed that two of the miRNA biogenesis-related genes *DCL1* and *SERRATE*, were significantly up-regulated in the embryogenic-induced explants of all three (*tpl*, *tpr1,* and *tpr4)* mutants (Figure 9). By contrast, there was a substantial increase of the *HEN1* transcripts exclusively in the *tpr4* mutant culture, which suggests that different TPL co-repressors might be involved in the control of distinct miRNA biogenesis genes in an embryogenic culture.

Taken together, the results showed that the TPL co-repressors might contribute to the AGL15-mediated negative control of the miRNA-biogenesis genes. Accordingly, AGL15 seems to cooperate with TPL, TPR1, and TPR to silence the *DCL1* and *SERRATE* genes while the cooperative interaction between AGL15 and TRP4 is assumed to repress *HEN1* during embryogenic induction.

## 3. Discussion

### 3.1. AGL15 Controls miR156 by Activating MIR156h Transcription during Embryogenic Induction

The presented transcript analysis of eight *MIR156* (*a–h*) genes in embryogenic cultures with different levels of *AGL15* expression (*agl15 agl18* vs. 35::*AGL15*) suggested that AGL15 might positively regulate the *MIR156h* expression during SE induction (Figure 1). Interestingly, *MIR156h* has also been indicated as being among the stress-regulated *MIR* genes [51] and the involvement of AGL15 in the regulation of stress-related genes has also been reported [52]. Therefore, we assumed that the activation of *MIR156h* by AGL15 during SE induction might be caused by the stress that is imposed by in vitro culture conditions including 2,4-D treatment [53]. In flower development, two other *MIR156* genes *MIR156a* and *MIR156c,* were directly activated by AGL15 [21] and together these findings imply that AGL15 might target different *MIR156* genes depending on the developmental process. Besides AGL15, other TFs have been reported to control individual *MIR156* genes including the involvement of FUS3 TF that activates *MIR156a* and *MIR156c* in seed development [54] and APETALA 2 TF that positively regulates *MIR156e* during flower development [55]. Whether these TFs, in particular FUS3, which have a substantial impact on embryogenic induction [43], might control the *MIR156* genes in an embryogenic culture remains to be explored.

The complexity of the TF-regulatory network that controls the *MIR156* gene expression has clearly been demonstrated in flower development. This network involves the direct activation of different *MIR156* genes by AP2 and AGL15 and the positive and direct regulation of *AGL15* by miR156-controlled AP2 [21,55]. The extensive regulatory interactions of AGL15 with different proteins, in particular, with other MADS proteins, such as AGL18, AGL24, and SHORT VEGETATIVE PHASE in the transcriptional control of the target genes have been demonstrated [13,21,56]. A number of MADS genes including *AGL18*, *AGL24*, *SVP* and *AP2*, which are differentially regulated in an embryogenic culture of Arabidopsis ([8,57]) provide candidates that can be used in the search for the proteins that cooperate with AGL15 in the transcriptional regulation of *MIR156h* in SE induction.

### 3.2. AGL15 Together with the TPL/TPR Corepressors Transcriptionally Repress the miRNA Biogenesis Genes DCL1, SERRATE and HEN1 during Embryogenic Induction

In addition to the transcriptional regulation, extensive posttranscriptional processing controls the activity of miRNA [58]. The complex and multi-layered regulation of the miRNA pathways results in frequent inconsistencies in the level of the pri- and mature miRNAs during plant development [37,51,59,60,61]. Similarly, the robust transcription of numerous *MIR* genes including *MIR156*s was associated with distinctly confined accumulation of the relevant mature miRNAs in an embryogenic culture of Arabidopsis [30].

The present results revealed that AGL15 is involved in the control of miR156 at different regulatory levels. In addition to regulating the pri-miR156 level, AGL15 was found to impact the production of mature miR156 in an embryogenic culture. Accordingly, AGL15 negatively affected the abundance of the mature miR156 that was produced by the different *MIR156* genes (Figure 2), which suggests a repressive role of AGL15 in the regulatory pathway that controls miR156 processing. The essential role of miRNA biogenesis genes in controlling embryogenic induction has also been demonstrated [31] and the *DCL1* gene, which has a central function in miRNA biogenesis, has been reported to be among the direct targets of AGL15 in an embryogenic culture of Arabidopsis [13]. In support of the involvement of AGL15 in miRNA processing, we found that AGL15 negatively affected the transcript level of *DCL1* and two other genes that are involved in miRNA biogenesis, *SERRATE,* and *HEN1* (Figure 3).

Complex and multi-layered regulatory interactions have been demonstrated to fine tune the components of the miRNA processing pathways [37,58,62]. However, our knowledge of the regulatory network that controls miRNAs remains incomplete and, in particular, the TFs that regulate the miRNA biogenesis genes in different tissues as well as the developmental processes need to be identified [37,58,62]. The present results indicated that AGL15 is a regulator of miRNA during the embryogenic transition that is induced in vitro in Arabidopsis. The study provides some evidence that AGL15 may control the miR156 activity by positively regulating the abundance of the pri-miR156 transcript and the negative control of the genes encoding crucial components of pri-miRNA biogenesis (*DCL1*, *SERRATE* and *HEN1*).

We hypothesised that the repressive function of AGL15 in miRNA biogenesis might also involve other miRNAs and in support of this, we found a substantially higher level of the mature miR167 and miR172 transcripts in the culture of the *agl15 agl18* mutant than in the 35S::*AGL15* line (Supplementary Figure S2). The role of AGL15 in the transcriptional regulation of miR172 has also been documented during flower development [21,55] and AGL15 has been shown to directly control the *MIR167* (*MIR167a*) expression in an embryogenic culture [52]. Importantly, a common histone acetylation-related mechanism controls the miR156, miR167 and miR172 biogenesis in Arabidopsis seedlings [63]. Hence, we postulate that similar to miR156, AGL15 might also repress miR167 and miR172 during SE, through an interaction with the histone deacetylases silencing complex. Further analysis is required to verify this hypothesis.

The results also imply that the AGL15 repression of the miRNA biogenesis genes in an embryogenic culture may involve the TPL/TPR co-repressors. Similarly, AGL15 in cooperation with TPL/TPR controls the genes that are involved in flowering [39]. An analysis of the embryogenic response of the *tpl/tpr* mutants (Figure 8) indicated that three of the corepressors, TPL, TPR2 and TPR4, control SE induction. Two of the SE-involved corepressors, TPL and TPR2, may directly interact with AGL15 through the TOPLESS domain, which has a binding affinity to the EAR domain that is present in numerous transcriptional repressors including AGL15 [39,64].

The results imply that different TPL/TPR proteins might control the different miRNA biogenesis genes during SE induction (Figure 9). Accordingly, TPL, TPR2 and TPR4 acting together seem to regulate the *DCL1* and *SERRATE* expression, while TPR4 alone may be involved in the repression of *HEN1*. The gene silencing complexes commonly consist of different TPL/TPR proteins [65,66]. However, recently, a monomeric form of TPL was found to be sufficient to cause an intense transcriptional repression in plants [67]. This finding supports our assumption that different TPL/TPR complexes, both composed and monomeric, might control the different miRNA biogenesis genes.

Besides cooperating with AGL15 to control the miRNA biogenesis genes other divergent and multiple roles of the TPL/TPR corepressors in SE induction might be expected because the TOPLESS proteins cooperate with a variety of TF families [39]. The representatives of these TF families, including WUS/WOXs, ARFs, AUX/IAA, and BBM, have an essential regulatory function during embryogenic induction [68,69,70,71,72,73]. Moreover, the essential function of TPL/TPRs in regulating the hormone (auxin)-responses including those that control the apical embryonic fate in zygotic embryos was found [40,64,74]. Thus, the versatile but as yet unknown regulatory functions of TPL/TPR in regulating the SE-transcriptome might be assumed and the contribution of these corepressors to the AGL15-controlled miRNA biogenesis seems to be one of them.

### 3.3. The AGL15-Mediated Repression of the miRNA Biogenesis Genes Involves Histone Deacetylation

The gene-repressive function of AGL15 involves the modulation of histone acetylation and a transcriptional repression motif in AGL15 recruits the histone deacetylase SIN3/HDAC complex components in order to repress the target genes [39,48]. We hypothesized that similar histone acetylation-related mechanisms operate during the AGL15-mediated repression of the miRNA biogenesis genes during embryogenic induction. In support of this assumption, we found that the repressive effect of AGL15 on the *DCL1*, *HEN1,* and *SERRATE* genes is modulated by TSA, which is an HDAC inhibitor (Figure 4) and that AGL15 significantly decreased the acetylation level of the H3 histone associated with the *DCL1* and *SERRATE* promoters in the embryogenic culture (Figure 7).

Although, in contrast to *DCL1* and *SERRATE*, the impact of AGL15 on the acetylation of histones in chromatin associated with *HEN1* promoter, was not found to be significant (Figure 7), the involvement of histone acetylation in the repression of *HEN1* in an embryogenic culture implies an increased expression of this gene in the *hdac* mutants (Figure 6). We cannot rule out the fact that another CArG motif located farther from TSS (−900 bp) might be involved in the histone acetylation-mediated and AGL15-controlled expression of *HEN1*. Different (de)acetylases and (de)methylases of histones interplay extensively in the control of gene expression and mutations in *HDAC19* may affect both the acetylation and methylation of histones [39,44,75,76,77,78]. Thus, histone methylation might contribute to the AGL15-mediated repression of *HEN1* and the role of histone methylation in the control miRNA biogenesis genes needs to be investigated in the future.

To learn more about the SIN3/HDAC components that are engaged in the repression of miRNA biogenesis genes during SE induction, we analysed the *HDAC6* and *HDAC19* deacetylases that have reported interactions with AGL15 [48]. A role of histone deacetylases including HDAC6 and HDAC19 in embryogenic development in Arabidopsis has been suggested [47,79]. Relevantly, we indicated that the *hdac6* and *hdac6/hdac19* mutations negatively affected the embryogenic response in the explant culture (Figure 5). In addition, the results offered some indirect clues on the involvement of HDAC6 and HDAC19 in the AGL15-mediated repression of the miRNA biogenesis genes during SE induction. Accordingly, we found that mutations in *HDAC6/19* positively affected the level of the *DCL1*, *SERRATE* and *HEN1* transcripts in the embryogenic culture (Figure 6). Importantly, HDAC19 works in conjunction with TPL to control the transition stage of zygotic embryogenesis [40]. The present results suggest a similar cooperative interaction of HDAC19 with TPL in repressing the miRNA biogenesis genes during embryogenic induction in somatic cells cannot be excluded (Figure 9).

Histone deacetylases interplay with histone acetylases to control gene expression including miRNA biogenesis genes [42,63,80]. HAG1/GCN5 histone acetylase contributes to the regulation of *DCL1* and *HYL1* in seedlings and its essential function in the acquisition of pluripotency in Arabidopsis has been indicated [81]. The role of GCN5 in the histone acetylation-mediated mechanism that controls the miRNAs in an embryogenic culture of Arabidopsis remains to be revealed.

## 4. Materials and Methods

### 4.1. Plant Material and Growth Conditions

Plants of *Arabidopsis thaliana* (L.) Heynh. Col-0 (WT) and two transgenic lines with opposite *AGL15* (AT5G13790) expression levels were used including the 35S::*AGL15* and *agl15 agl18* double mutant. In addition, the insertional mutants in the *TPL* and *HDAC* genes (*tpl*—N68599, *tpr1*—N522964, *tpr3*—N529936, *tpr4*—N502209, *hdac6* and *hdac6 hdac19*) were studied. The 35S::*AGL15* was kindly provided by Sheryn Perry (University of Kentucky, Lexington, KY, USA), the *agl15 agl18* mutant by Donna E. Fernandez (Department of Botany, University of Wisconsin, Madison, WI, USA) and the *hdac6* and *hdac6 hdac19* by Kim Boutilier (Wageningen University & Research, Wageningen, Netherlands). The seeds of the other genotypes that were analysed were purchased from NASC (The Nottingham Arabidopsis Stock Centre, Nottingham University, Nottingham, UK). The seeds were sown in 42-mm-diameter Jiffy-7 peat pots (Jiffy) and the plants were grown in a “walk-in” type phytotron under controlled conditions: 22 °C, 16 h/8 h (light/dark) and a light intensity of 100 µE/m^2^s. The cultures that were grown in vitro were maintained in a controlled growth chamber at 22 °C, 16 h/8 h (light/dark) and a light intensity of 50 µE/m^2^s.

### 4.2. Somatic Embryogenesis Induction In Vitro

Immature zygotic embryos at the late cotyledonary stage were used as the explants for the in vitro cultures according to the standard protocol [82]. The explants were excised from the siliques 10–12 days after pollination, sterilised with sodium hypochlorite (20% commercial bleach) and washed thoroughly with sterile water. The explants were cultured on an E5 solid medium containing B5 salts and vitamins [83] that had been supplemented with 5.0 µM 2,4-D (2,4-dichlorophenoxyacetic acid, Sigma, St. Louis, MO, USA), 20 g L^−1^ sucrose and 8 g L^−1^ agar (Oxoid, Hampshire, UK). The explant capacity for SE was evaluated in a three-week-old culture and two parameters were evaluated including SE efficiency, i.e., the percentage of the explants that had formed somatic embryos and SE productivity, i.e., the average number of somatic embryos that had been produced by embryogenic explant.

### 4.3. Analysis of the Mature miRNA and Target Gene Expression

Total RNA was isolated from the explants that had been cultured for 0, 5 and 10 d on the SE-induction E5 medium. In some experiments, the E5 medium was supplemented with 1.0 µM of trichostatin A (TSA), which is a blocker of the HDAC activity. RNA was isolated using a miRVana miRNA Isolation Kit. Depending on the age of the culture, 250 (0 d) to 50 (10 d) explant-derived cultures were used for the RNA isolation in one biological replicate. The concentration and quality of the isolated RNA was evaluated using an ND-1000 NanoDrop spectrophotometer (Nano Drop Technologies, LLC, Wilmington, DE, USA). The miRNA-specific and oligo-dT primers and RivertAid First Strand cDNA synthesis kit (Thermo Fisher Scientific, Waltham, MA, USA) were used to synthesise the cDNA. The mature miRNAs were identified according to Speth and Laubinger [84]. The product of the reverse transcription was diluted with water at a 1:4 ratio and 2.0 µL of the solution was used for the Real-Time RT qPCR. LightCycler Fast-Start DNA Master SYBR Green I (Roche, Basel, Switzerland) and the primers that were relevant to the genes being studied were used to determine the Real-Time RT qPCR reactions (Supplementary Table S1 [85,86]). The Cp values were calculated using LinRegPCR software. The relative RNA and miRNA levels were calculated and normalised to an internal control, the *At4g27090* gene encoded 60S ribosomal protein. The control gene had a constant expression pattern (C_T_ = 18 ± 1) in all of the tissue samples that were analysed. The plant tissues for the real-time (RT) qPCR analysis were produced in three biological repetitions and two technical replicates of each repetition were carried out. The relative expression level was calculated using 2^−∆∆*C*T^, where ∆∆C_p_ represents ∆C_T_^reference condition^ − ∆C_p_^compared condition^.

### 4.4. Chromatin Immunoprecipitation (ChIP)

The Yelagandula et al. ChIP method [87] was used with some modifications. The conditions of de-/crosslinking and chromatin shearing were optimised for the plant material being analysed. Chromatin extracts were prepared from the cultured explants (~50 mg of tissue per ChIP) and then treated with 1% formaldehyde for 20 min on ice under a vacuum. The chromatin was sheared to a mean length of 500 bp by sonication (Bioruptor^®^ Plus, Diagenode, Denville, NJ, USA) and the complex of proteins and DNA fragments was then immunoprecipitated using the polyclonal antibodies against the acetylated forms of histone H3 (2 µg; Merck, St. Louis, MO, USA, Cat. no. 06-599). The DNA that was cross-linked to the immunoprecipitated proteins was reversed and analysed using qPCR and the gene-specific primers. A LightCycler 480 (Roche, Basel, Switzerland) real-time detection system was used to analyse the relative acetylation level of the chromatin associated with *DCL1*, *HEN1,* and *SERRATE* genes. The qPCR reaction was performed as described by [84]. The primers that were used in the qPCR were designed using Primer3Plus software (version 3, Molbi, Michelstand, Germany) (Supplementary Table S2). The genomic sequences that were analysed in the *DCL1, HEN1,* and *SE* genes were localised in the TSS + 300 bp region and included the AGL15-binding CArG motif (Supplementary Figure S1). The Cp values were calculated using LinRegPCR software (version 11, Academic Medical Centre, Amsterdam, The Netherlands). The ChIP-qPCR data were normalised using the percent input method. The H3ac level is presented as 2^(adjusted input—Cp (xx gene)^ × 100%. Three biological replicates and two technical replicates were analysed for each combination.

### 4.5. Statistical Analysis

The Student’s *t*-test (*p* < 0.05) or a two-way ANOVA (*p* < 0.05) followed by Tukey’s honestly significant difference test (Tukey HSD-test) (*p* < 0.05) were used to calculate any significant differences between the combinations. The graphs show the averages with the standard deviation (SD); the statistical analysis was performed using the medians.

## 5. Conclusions

**Figure 10:**
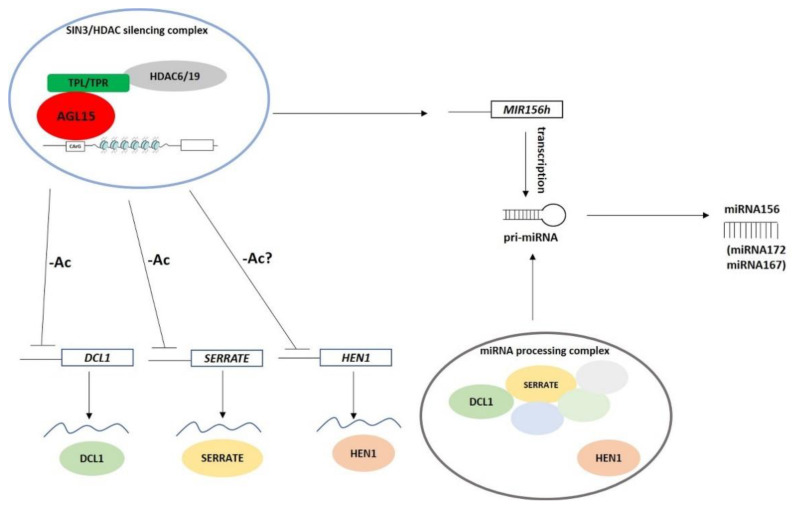
A putative model of the histone acetylation-related and AGL15-mediated control of miR156 during embryogenic induction. AGL15 is postulated to fine tune the abundance of miR156 by activating the *MIR156* gene (*MIR156h*) expression and the containment of the mature miR156 molecules as a result of the repression of the miRNA biogenesis genes *DCL1*, *SERRATE* and *HEN1.* To repress the target genes, AGL15 interacts with the TOPLESS co-repressors (TPL, TPR1, TPR4) and recruits the histone deacetylases, HDAC6 and HDAC19, which are components of the SIN3/HDAC gene silencing complex [39,48]. The AGL15-mediated histone deacetylation might also control other miRNAs including miR167 and miR172 in embryogenic culture. AGL15—AGAMOUS-like15, DCL1—DICER-like1, HEN1-HUA-ENHANCER1, TPL—TOPLESS, TPR—TOPLESS-RELATED, HDAC—HISTONE DEACETHYLASE.

The study demonstrated that AGL15 might control the abundance of miR156 during embryogenic induction through diverse regulatory pathways that involve the positive transcriptional regulation of *MIR156* (*MIR156h*) and the containment of the abundance of mature miR156 via the downregulation of the miRNA biogenesis genes *DCL1, SERRATE,* and *HEN1* (Figure 10).

Two histone deacetylases, HDAC6 and HDAC19, were postulated to contribute to the AGL15-mediated repression of miRNA biogenesis during somatic embryogenesis. However, histone acetylation might also regulate the transcription of the *MIR* genes including *MIR156* [88]. Given that HDACs can also function as transcriptional activators of genes [89,90], the role of HDAC6/19 in the AGL15-mediated activation of the *MIR* genes (e.g., *MIR156h*) might also be expected. In support of such a scenario, a histone acetyltransferase, GCN5, besides repressing miRNA processing, was required for the expression of a subset of the *MIR* genes [63]. Interestingly, HDAC19 interacts with the transcriptional activator of the auxin-responsive genes specifically in the cells that have an elevated auxin concentration [91]. This finding suggests that HDAC19, similar to GCN5 [63], might control the abundance of miRNA in an auxin-induced embryogenic culture through the repression of a subset of the miRNA biogenesis genes and the activation of the *MIR* genes. The *MIR*-regulatory function of *HDAC19* requires experimental verification.

Histone (de)acetylases may also regulate miRNA processing through changes in the acetylation status of the regulatory proteins of the microprocessor complex [92,93]. Whether histone acetylases/deacetylases (e.g., HDAC6 and 19) affect the proteins that control the miRNA pathways in an embryogenic culture remains to be revealed.

In conclusion, the present study indicates that similar to mouse and human cells [94,95], histone acetylation controls the miRNA pathways during the reprogramming of plant somatic cells to pluripotency and that AGL15 seems to play an essential regulatory role in this mechanism.

## Supplementary Materials

Can be found at https://www.mdpi.com/1422-0067/21/18/6733/s1. Supplementary Figure S1. Localisation of the CArG sequence in the promoter region of the *DCL1* (A), *SERRATE* (B) and *HEN1* (C) genes. Green boxes indicate the presence of a CArG sequence and orange lines indicate the sequence that was amplified during the Real Time qPCR analysis after the ChIP analysis. Supplementary Figure S2. Level of mature miR172 and miR167 in the embryogenic cultures of the 35S::*AGL15* and *agl15 agl18* transgenic lines. The relative miRNA level was normalised to an internal control (*At4g27090*) and calibrated to the Col-0 culture of the same age. * value significantly different from the 35S::*AGL15* culture of the same age (*p* < 0.05; *n* = 3 ± SD). Supplementary Table S1. Primer’s sequence used for gene expression analysis. Supplementary Table S2. Primer’s sequences used in the ChIP analysis.

## Abbreviations

AGL15	AGAMOUS-like 15
SE	somatic embryogenesis
TF	Transcription factor
TSS	Transcription start site
miRNA	microRNA
*DCL1*	*DICER-like1*
*HEN1*	*HUA-ENHANCER1*
*HYL1*	*HYPONASTIC LEAVES1*
*TPL*	*TOPLESS*
*TPR*	*TOPLESS-RELATED*
*HDAC*	*HISTONE DEACETYLASE*
TSA	Trichostatin A
ChIP	Chromatin immunoprecipitation
SIN3/HDAC	SWI-INDEPENDENT3/HISTONE DEACETYLASE
