# Supplementary material for: AGL15 Controls the Embryogenic Reprogramming of Somatic Cells in Arabidopsis through the Histone Acetylation-Mediated Repression of the miRNA Biogenesis Genes
Source: Int J Mol Sci. 2020 Sep 14;21(18):6733. doi: 10.3390/ijms21186733 (PMC7554740; doi:10.3390/ijms21186733)
Supplement: Supplementary file 1 [file ijms-21-06733-s001.pdf]

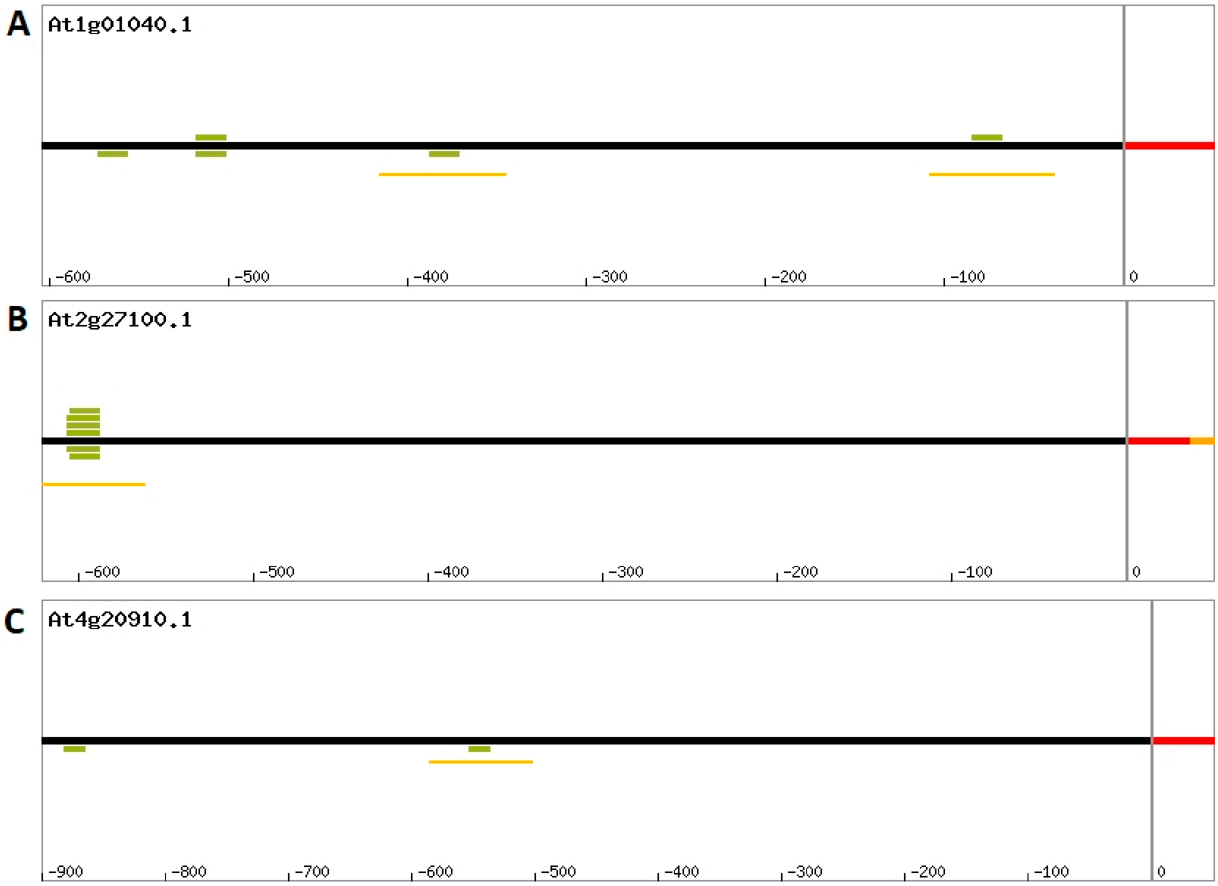

Supplementary Figure S1. Localisation of the CArG sequence in the promoter region of the *DCL1* (**A**), *SERRATE* (**B**) and *HEN1* (**C**) genes. Green boxes indicate the presence of a CArG sequence and orange lines indicate the sequence that was amplified during the Real Time qPCR analysis after the ChIP analysis.

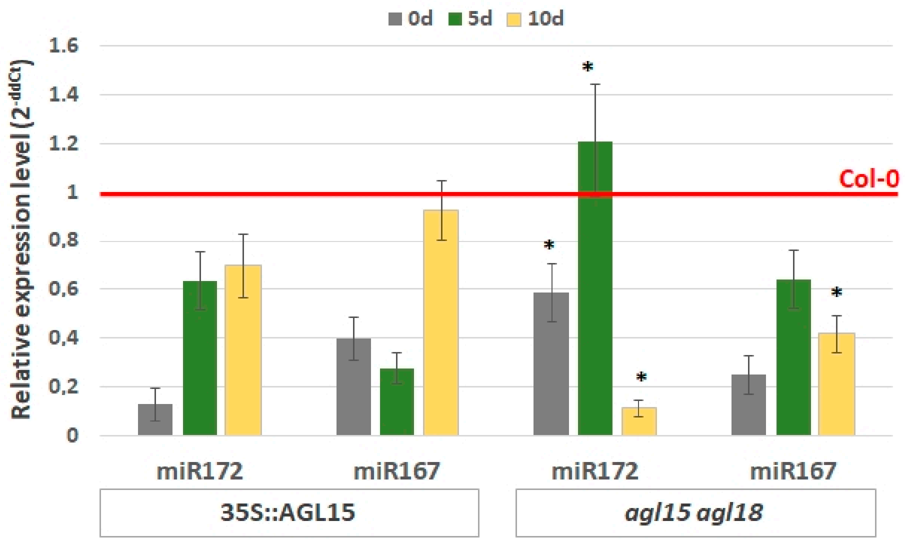

Supplementary Figure S2. Level of mature miR172 and miR167 in the embryogenic cultures of the 35S::*AGL15* and *agl15 agl18* transgenic lines. The relative miRNA level was normalised to an internal control (*At4g27090*) and calibrated to the Col-0 culture of the same age. * value significantly different from the 35S::*AGL15* culture of the same age ($p < 0.05$; n = 3 ± SD).

Supplementary Table S1. Primer's sequence used for gene expression analysis.

Gene	Primer sequence
At4g27090	[85]
miR156a-f	[30]
miR156g	[30]
miR156h	[30]
miR172	[30]
DCL1	[30]
HEN1	[30]
SERRATE	[30]
AGL15	[86]
HYL1	F-GTGCCAGAAGGTCGAAACTC<br>R - TTCTTGTTCAGCTCCTGTG
miR167	F- TTCCTTGATTGAGCCGCGCC<br>R - GTGCAGGGTCCGAGGT
TPL	F – TATGGAAATGGCAGCGGAATGAGC<br>R - GCGGTAAAGAAGCTGTCGCCTTTC
TPR1	F ACCTTCGATTGCGTTGCAGAGTC<br>R - TGGATGGACATTCGAGTTGCTGAG
TPR2	F -TGCCTCAACCTACACCAAACAGTG<br>R - TCGGTTCTTGTGGATGAGCTGTG
TPR3	F – AGCAGAACCCTAGTGGAAAGGC<br>R - CTGTTTGGTTGCCAATGCTGAGG
TPR4	F – TTGTCCATGCCAGCCAGTTAGC<br>R – CTTGACTCTTTCGGTATCCACTGC
HDAC6	F – AACCTCGCATCTGGAGTGGAAC<br>R - ATCTTCACCGGTAGAGTCCCTGTC
HDAC19	F – TCTTGGGTGGTGGTGGTTACAC<br>R - TCCAAGTGCAACTCCAGTCTCG

Supplementary Table S2. Primer's sequences used in the ChIP analysis.

Fragment of promoter	Sequence
DCL1 TSS +300	F- TTGCTCTTCTTCGTGACCCC<br>R -AGGGGTGAGAGAAGAAGAGTGT
SERRATE TSS + 300	F- TGGAGGAATAGTCAAAGGTGTGG<br>R - AAGCCTCTGGTGGTCTCTCCCT
HEN1 TSS + 300	F- TGTCAAACGATCAGGAAAGATTGG<br>R- TGGCTCTGATTTGAATTGTCAAGT
DCL1 fragment 1	F – GTTTAGAACCAACCAATCAC<br>R –GTTTTACGACTTATTCCACGC
DCL1 fragment 2	F – CAAAGTCGAATAAATTATTTACC<br>R –TTGCTTGCTTCTGTTTTGTAG
SERRATE fragment 1	F - AGGAGAGAGGTGGTCTGTGTC<br>R - CTCCTCTCGCATAAGATTCCT
